# Spatially microdissected profiling of tonsillar germinal centers reveals a KLRG1-enriched immune signature in IgA nephropathy

**DOI:** 10.3389/fimmu.2026.1750300

**Published:** 2026-05-22

**Authors:** Mayuko Kawabe, Izumi Yamamoto, Yutaro Ohki, Ayaka Hayashi, Go Kanzaki, Kei Matsumoto, Hiroyuki Ueda, Keita Hirano, Nobuo Tsuboi, Takashi Yokoo

**Affiliations:** Division of Nephrology and Hypertension, Department of Internal Medicine, The Jikei University School of Medicine, Tokyo, Japan

**Keywords:** germinal center, IgA nephropathy, KLRG1, mucosal immunity, tonsil

## Abstract

**Background:**

Geographic variability in the clinical efficacy of tonsillectomy for immunoglobulin A nephropathy (IgAN) suggests population-level differences in mucosal immune architecture. However, the molecular features of the tonsillar germinal center (GC) microenvironment—the central site for IgA class switching and mucosal B-cell activation—remain poorly characterized, particularly in relation to glomerular injury severity.

**Methods:**

In this exploratory case–control study, we analyzed tonsils from biopsy-proven IgAN (n=5) and matched habitual tonsillitis controls (n=5). Laser microdissection was used to isolate GC plus mantle zone and non-GC regions, followed by immune transcriptomic profiling using the Nanostring Human Immunology V2 Panel. Differential expression was assessed with FDR correction. KLRG1 expression patterns were validated by immunohistochemistry.

**Results:**

Laser microdissection revealed a GC-enriched immune signature in IgAN, characterized by upregulation of KLRG1 (fold change 3.2, p < 0.001) and ZBTB16, alongside downregulation of CCL18, FADD, and TNFRSF17. Immunohistochemistry demonstrated GC-localized KLRG1 expression in IgAN, which appeared more evident in cases with crescentic glomerular lesions. Non-crescentic IgAN showed marginal GC localization, while controls exhibited predominantly extrafollicular KLRG1 expression. Whole-tissue analysis supported enrichment of innate-like lymphocyte–associated genes, consistent with a mucosal activation–associated pattern.

**Conclusion:**

This study identifies a spatially defined, KLRG1-enriched GC signature in IgAN. These findings suggest a potential association between GC-localized KLRG1 expression and glomerular injury severity; however, given the exploratory nature and small sample size, they should be considered hypothesis-generating. Further studies are required to validate these observations.

## Introduction

Immunoglobulin A nephropathy (IgAN) is the most prevalent primary glomerulonephritis worldwide and a major cause of end-stage kidney disease (1). A central pathogenic feature is the overproduction of galactose-deficient IgA1 (Gd-IgA1) at mucosal immune sites, followed by mesangial deposition and glomerular inflammation (2–4). Among mucosal lymphoid tissues, nasal-associated lymphoid tissue (NALT) and gut-associated lymphoid tissue (GALT) have been identified as major sources of nephritogenic IgA1 (5–8). In Japan, tonsillectomy—with or without corticosteroid therapy—has been widely adopted for IgAN, with several studies demonstrating clinical benefit (9–14), whereas Western cohorts have shown inconsistent results (15, 16). These discrepancies raise the possibility that regional differences in mucosal immune architecture, including germinal center (GC) biology, influence treatment responses.

The tonsillar GC microenvironment is the principal site of IgA class switching, affinity maturation, and APRIL- and BAFF-mediated B-cell selection (17–21). However, most prior studies have analyzed whole tonsillar tissue, limiting insight into GC-specific immune processes. Understanding GC-resident immune phenotypes is therefore critical for clarifying how mucosal dysregulation contributes to systemic Gd-IgA1 production and downstream kidney injury. Whether specific GC immune features distinguish IgAN from non-IgAN tonsillar inflammation, and whether such features relate to glomerular injury severity, remains largely unknown.

KLRG1 is a well-established marker of terminally differentiated effector T cells, CD8^+^ cytotoxic lymphocytes, and innate-like subsets, which are typically excluded from germinal center niches. Therefore, the unexpected enrichment of KLRG1^+^ cells within the tonsillar GC suggests a profound alteration in the selection environment that could reshape IgA class switching and affinity maturation dynamics. These considerations raise the possibility that IgAN harbors a unique mucosal activation phenotype driven by aberrant GC remodeling, which has not been previously characterized. Given these observations, a spatially resolved approach is required to accurately define the immune architecture of the tonsillar germinal center in IgAN.

Spatial transcriptomics and laser microdissection now make it possible to interrogate defined immune niches with high molecular resolution (22). These approaches provide a unique opportunity to uncover disease-specific GC signatures that may explain geographic variation in tonsillectomy efficacy and refine mechanistic models of IgAN. Accordingly, this study aimed to characterize the spatial immune architecture of the tonsillar GC in IgAN and to explore its relationship with glomerular injury severity.

## Materials and methods

### Study design and participants

This exploratory, case–control study included patients undergoing elective tonsillectomy at the Jikei University School of Medicine, Tokyo, Japan, between 2000 and 2021. The IgAN group comprised five patients with biopsy-proven IgAN, diagnosed according to the 2016 Oxford classification (23) based on segmental or diffuse granular mesangial IgA deposits on immunofluorescence microscopy. Exclusion criteria were: (1) prior corticosteroid or immunosuppressive therapy within 6 months before surgery, (2) concurrent systemic autoimmune disease, (3) active infection at the time of surgery, and (4) insufficient tonsillar tissue for analysis. Controls were five patients with habitual tonsillitis, matched to the IgAN group by age (± 5 years), sex, serum creatinine level (± 0.2 mg/dL), and year of surgery. Clinical and pathological characteristics were extracted from medical records and summarized in **Table 1**. Given the exploratory nature and the rarity of surgically obtained tonsils from biopsy-proven IgAN, a formal sample size calculation was not feasible; thus, this study was designed as a hypothesis-generating analysis.

**Table 1:** Baseline clinical and pathological characteristics of patients with habitual tonsillitis and immunoglobulin A nephropathy (IgAN).

Characteristic	Habitual tonsillitis (N = 5)	IgA nephropathy (N = 5)	P value
Age (year)	35.2 ± 2.9	39.2 ± 3.9	0.10
Sex male, n (%)	3 (60)	3 (60)	1.00
Serum Creatinine (mg/dL)	0.76 ± 0.17	0.83 ± 0.23	0.59
eGFR (mL/min/1.73m²)	86.2 ± 9.9	77.2 ± 12.2	0.24
Proteinuria (g/gCr)	(-)	0.59 ± 0.28	
Hematuria, n (%) Negative or ± +1 +2 +3	Negative or ±, 5 (100) +1, 0 (0) +2, 0 (0) +3, 0 (0)	Negative or ±, 0 (0) +1, 0 (0) +2, 3 (60) +3, 2 (40)	0.007
Oxford Classification		M0E0S0T0C1 M0E0S1T0C1 M0E0S1T0C1 M0E0S1T0C0 M0E1S1T0C0	

Values are presented as mean ± standard deviation or number (percentage), unless otherwise indicated. Proteinuria was not detected in patients with habitual tonsillitis. Hematuria severity was graded by dipstick as negative/±, +1, +2, or +3. Oxford classification parameters (M, mesangial hypercellularity; E, endocapillary hypercellularity; S, segmental glomerulosclerosis; T, tubular atrophy/interstitial fibrosis; C, cellular or fibrocellular crescents) are shown for each IgAN patient.

eGFR, estimated glomerular filtration rate; g/gCr, grams of protein per gram of creatinine.

### Tissue processing and immunohistochemistry

Tonsil specimens were formalin-fixed, paraffin-embedded (FFPE), and sectioned at 4 µm thickness. Hematoxylin–eosin staining was performed for histological assessment. Immunohistochemistry was conducted using antibodies against CD3, CD4, CD8, CD10, CD20, CD138, IgA, galactose-deficient IgA1 (KM55, 1:10; Immuno-Biological Laboratories), and killer cell lectin-like receptor G1 (KLRG1; 1:100; Sigma-Aldrich, HPA076494). The EnVision™+ System (Dako) with diaminobenzidine chromogen was used for visualization. Two blinded observers independently evaluated the staining patterns, recording the presence/absence and predominant localization (GC plus mantle zone vs. non-GC regions).

### Laser microdissection and RNA extraction

Two consecutive 10 µm FFPE sections from each specimen were subjected to laser microdissection using a Leica LMD6500 system. Regions of interest included (1) the GC plus mantle zone and (2) the non-GC regions. RNA was extracted using the NucleoSpin^®^ totalRNA FFPE XS Kit (TaKaRa Bio) per the manufacturer’s instructions. RNA purity was assessed by NanoDrop 2000 spectrophotometry (Thermo Fisher Scientific), with acceptable quality defined as A260/280 ratio 1.7–2.3 and A260/230 ratio 1.8–2.3. Due to FFPE-related degradation, RNA Integrity Number (RIN) was not determined. Nonetheless, Nanostring technology is largely insensitive to RNA fragmentation, and reliable gene expression measurements can be obtained even when RIN values are not measurable in FFPE samples.

### Gene expression profiling

Expression of 579 immune-related genes was quantified using the nCounter^®^ Human Immunology V2 Panel (Nanostring Technologies). A total of 250 ng of total RNA was used per reaction. Hybridization, post-hybridization processing, and digital counting were performed according to the manufacturer’s instructions. Data quality control, background subtraction, and normalization to the geometric mean of housekeeping genes were performed using the ROSALIND^®^ platform (OnRamp Bioinformatics). Quality control metrics were assessed based on NanoString positive and negative control probes, and all samples passed quality control and were included in downstream analyses.

### NanoString sample-level expression analysis

Normalized NanoString expression data were analyzed at the individual sample level. Expression values were log2-transformed prior to analysis. Key genes of interest (KLRG1, ZBTB16, CCL18, FADD, and TNFRSF17) were visualized using paired plots comparing the GC plus mantle zone and non-GC regions within each case. Heatmap visualization was performed using row-wise z-score normalization (per gene) to compare relative expression patterns across samples. Principal component analysis (PCA) was conducted using all genes based on log2-transformed normalized expression values without additional scaling. To ensure that the observed patterns were not driven by individual samples, we examined consistency across samples using paired plots, heatmap visualization, and PCA.

### Quantification of KLRG1 immunohistochemistry

KLRG1 immunohistochemical staining was quantitatively analyzed using Fiji (ImageJ, NIH, USA). Whole-slide images were captured under identical acquisition conditions. For each case, multiple regions of interest (ROIs) were systematically selected from the GC plus mantle zone and non-GC regions based on predefined histological criteria. Multiple GC areas were included per case where available. Color deconvolution (H DAB) was applied to separate the DAB signal. The DAB channel was used for subsequent quantification. A fixed threshold was applied uniformly across all samples to identify KLRG1-positive signals. The threshold was determined empirically based on representative images and then kept constant for all analyses to ensure comparability. Within each ROI, the KLRG1-positive area was calculated as the percentage of DAB-positive pixels relative to the total ROI area (% area). Multiple ROIs were analyzed per region in each case, and the mean value per case was used for statistical analysis. To minimize technical artifacts, ROIs containing staining artifacts (e.g., edge effects, tissue folds, or nonspecific staining) were excluded based on morphological assessment. This ROI-level exclusion was performed prior to averaging. Quantification was performed using a consistent threshold across all samples to ensure comparability of staining intensity. Typically, 4–6 ROIs were selected per region per case, including multiple germinal centers where available. Regions of interest were defined based on CD10 and CD20 immunostaining patterns, with GC plus mantle zone areas identified by characteristic CD10-positive follicular structures and surrounding CD20-positive B-cell zones. Non-GC regions were defined as CD10-negative interfollicular areas outside follicular structures. ROI selection was independently confirmed by two blinded observers.

### Statistical analysis

Continuous variables were expressed as mean ± standard deviation (SD) and compared between groups using unpaired t-tests. Categorical variables were compared using χ² or Fisher’s exact test, as appropriate. For gene expression data, differential expression between groups was assessed using Student’s t-test with Benjamini–Hochberg false discovery rate (FDR) correction for multiple testing. Genes with FDR-adjusted p < 0.05 were considered statistically significant. Effect sizes were reported as fold change, given the small sample size. For quantitative image analysis of KLRG1 immunohistochemistry, per-case values were used for group comparisons between IgAN and habitual tonsillitis, and statistical significance was assessed using the Mann–Whitney U test. The raw Nanostring data generated in this study have been deposited in the Zenodo repository and are publicly available at https://zenodo.org/records/17111571. All other data supporting the findings of this study are available from the corresponding author upon reasonable request.

## Results

### Clinical characteristics

Baseline characteristics of the IgAN and habitual tonsillitis groups are summarized in **Table 1**. The IgAN group (n = 5; 3 males, 60%) had a mean age of 39.2 ± 3.9 years and mean serum creatinine of 0.83 ± 0.23 mg/dL, with mean proteinuria of 0.59 ± 0.28 g/gCr. Three patients (60%) exhibited crescent formation on kidney biopsy. The habitual tonsillitis group (n = 5; 3 males, 60%) had a mean age of 35.2 ± 2.9 years and mean serum creatinine of 0.76 ± 0.17 mg/dL. There were no significant differences between groups in age, sex, or renal function. Although the groups were well matched, the small sample size warrants cautious interpretation.

### Immunohistochemical analysis

**Figure 1:**
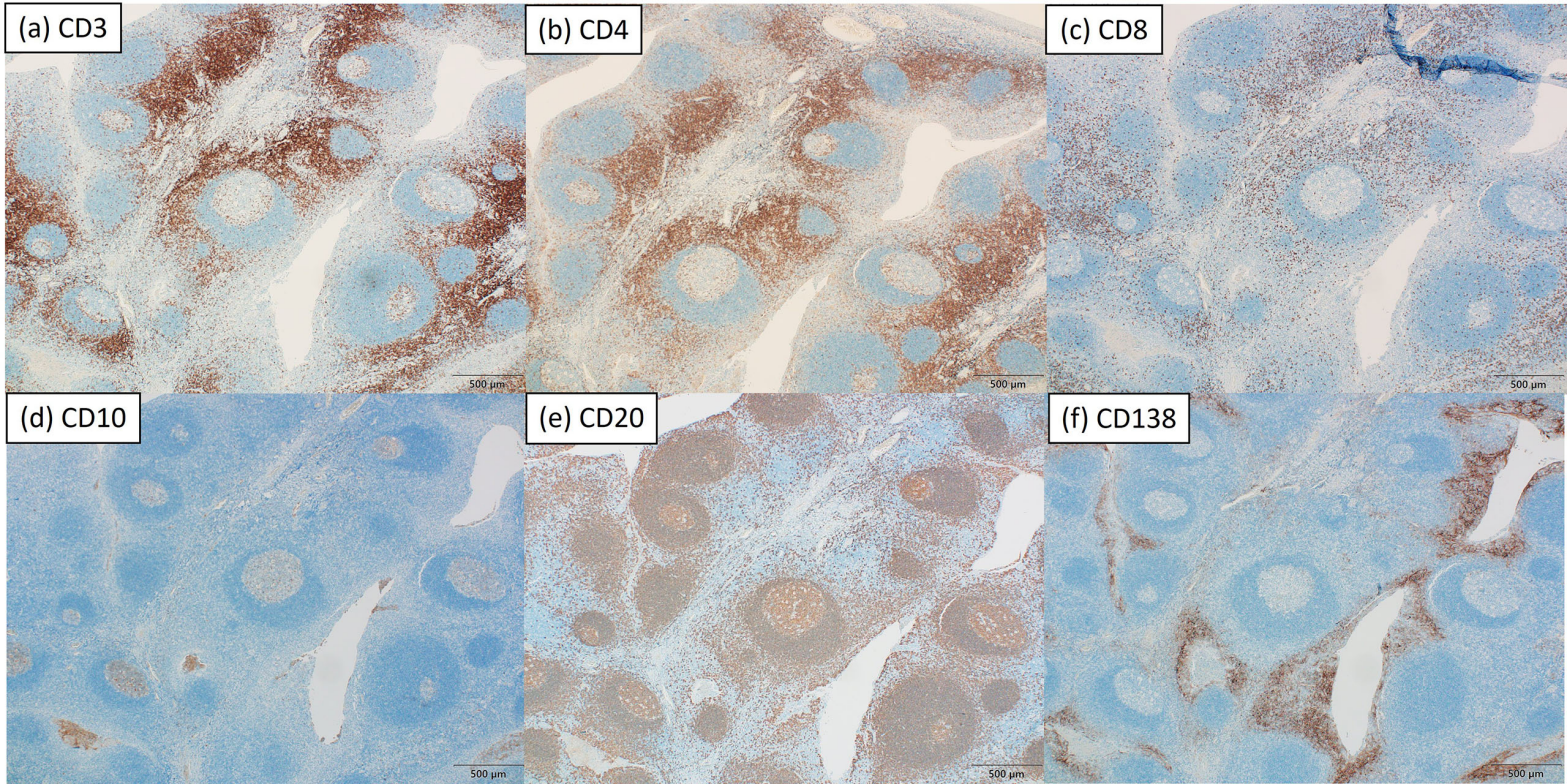
Immunohistochemical staining of a tonsil from a representative patient with IgA nephropathy. Representative immunohistochemical staining for CD3 **(a)**, CD4 **(b)**, CD8 **(c)**, CD10 **(d)**, CD20 **(e)**, and CD138 **(f)** in serial sections of the same tonsillar specimen (magnification ×40). CD10 and CD20 were localized predominantly in the GC plus mantle zone, whereas CD3, CD4, and CD8 were broadly distributed in non-GC regions. CD138 staining was confined mainly to crypt epithelium. Scale bar: 500 μm.

**Figure 2:**
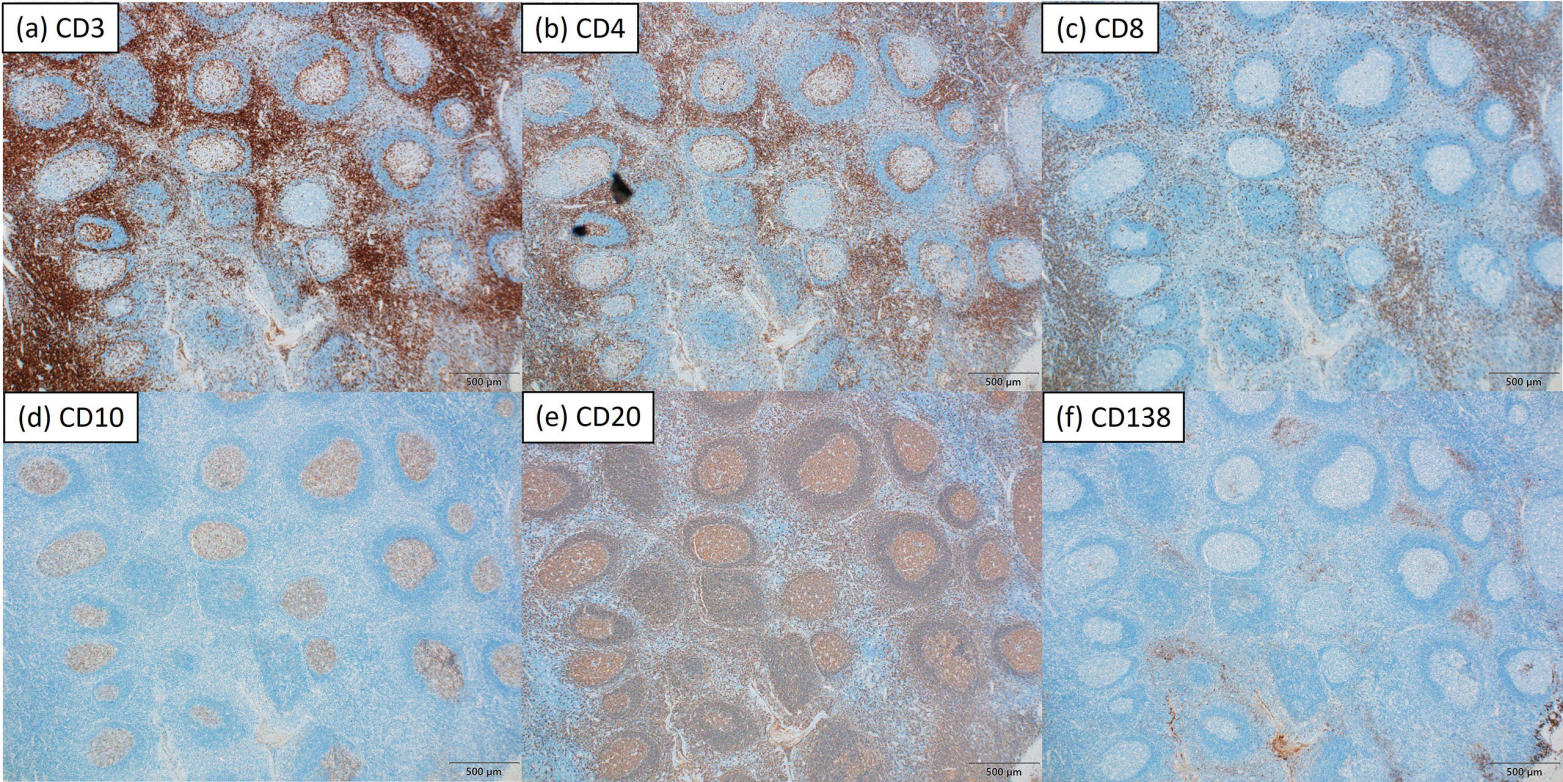
Immunohistochemical staining of a tonsil from a representative patient with habitual tonsillitis. Representative immunohistochemical staining for CD3 **(a)**, CD4 **(b)**, CD8 **(c)**, CD10 **(d)**, CD20 **(e)**, and CD138 **(f)** in serial sections of the same tonsillar specimen (magnification ×40). The overall distribution patterns of these markers were comparable to those observed in IgA nephropathy. Scale bar: 500 μm.

**Figure 3:**
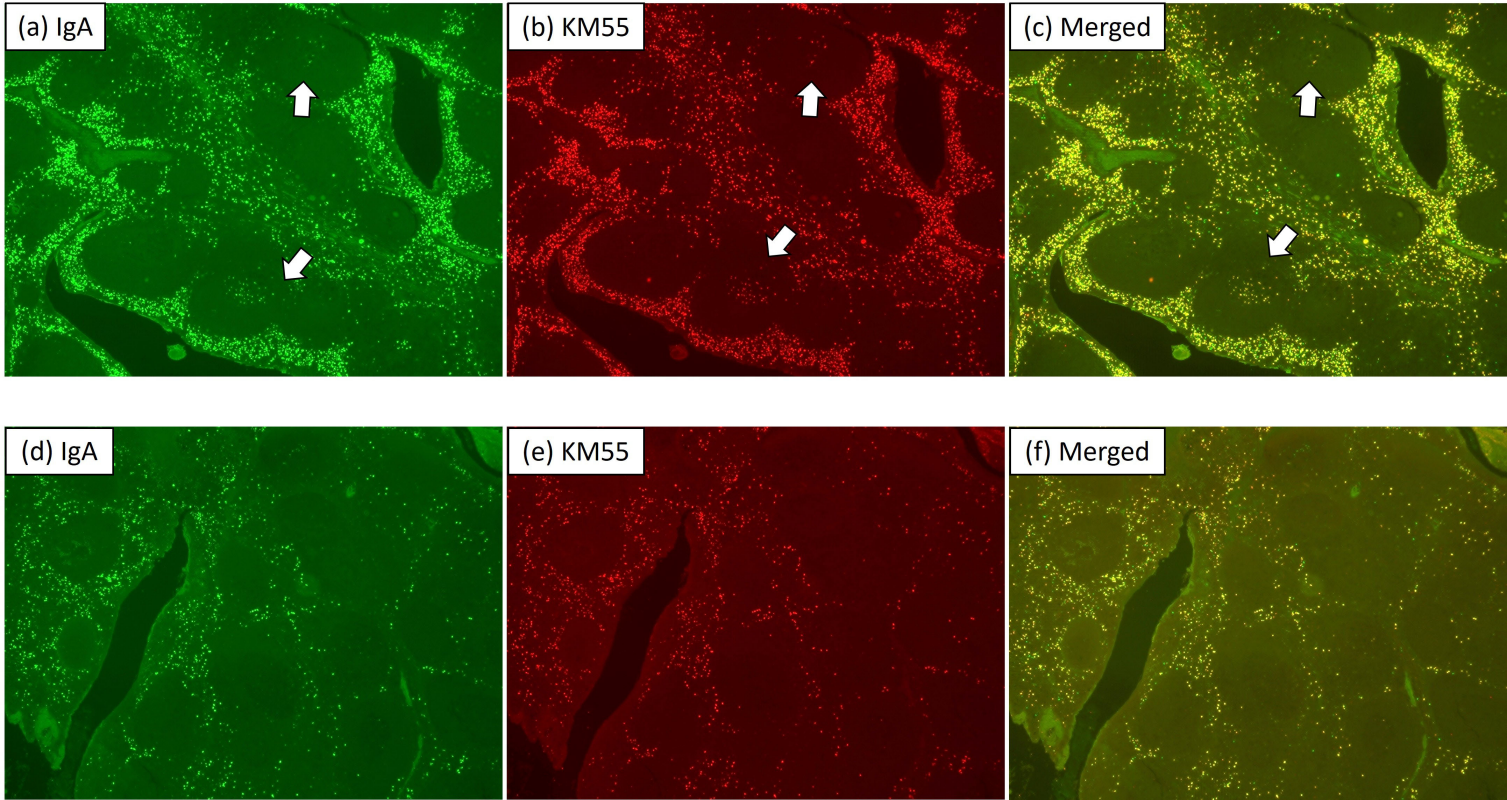
IgA and galactose-deficient IgA1 staining in tonsils from IgA nephropathy and habitual tonsillitis. Representative images of IgA **(a, d)**, Gd-IgA1 (KM55) **(b, e)**, and merged staining **(c, f)** in IgA nephropathy (upper panels) and habitual tonsillitis (lower panels) (magnification ×40). IgA and Gd-IgA1 deposition was more frequent and intense within germinal centers of IgA nephropathy tonsils (arrows) compared with controls, highlighting enhanced mucosal IgA activity in IgAN.

CD3, CD4, CD8, CD10, CD20, and CD138 showed comparable distribution patterns between groups (**Figures 1**, **2**). CD10 and CD20 were predominantly localized in the GC plus mantle zone, whereas CD3, CD4, and CD8 were more broadly distributed in non-GC regions. CD138 was confined mainly to crypt epithelium. IgA and Gd-IgA1 (KM55) depositions were more frequent and intense in IgAN tonsils, particularly within germinal centers, compared with controls (**Figure 3**).

### Transcriptomic profiling of the germinal center plus mantle zone

**Figure 4:**
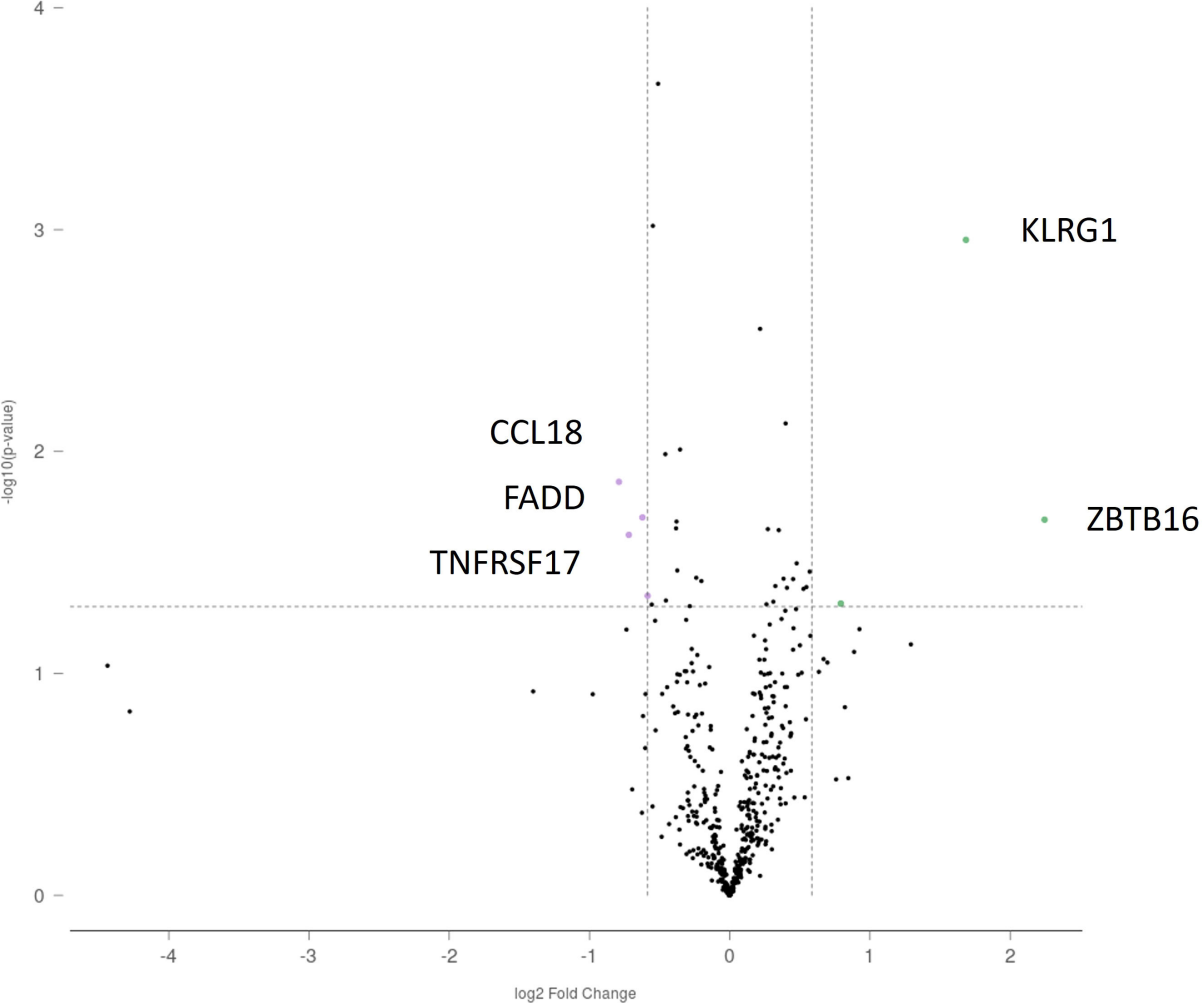
Differentially expressed genes in the germinal center plus mantle zone between IgA nephropathy and habitual tonsillitis. Volcano plots illustrating differential gene expression in laser-microdissected GC plus mantle zones. KLRG1 and ZBTB16 showed higher expression in IgA nephropathy, whereas CCL18, FADD, and TNFRSF17 were downregulated after FDR correction. These findings indicate a distinct germinal center–enriched immune signature in IgAN.

**Figure 5:**
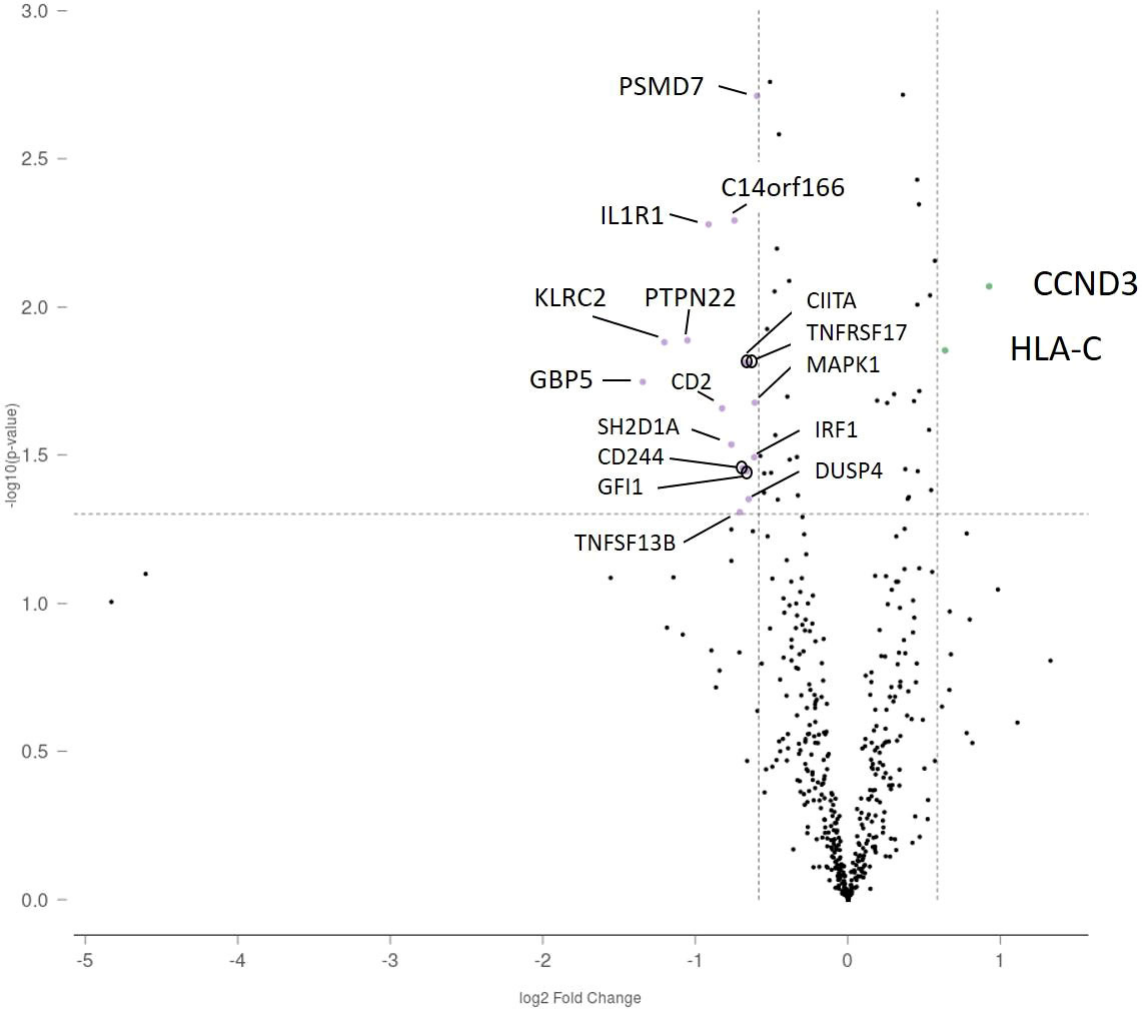
Differentially expressed genes in the non-germinal center region between IgA nephropathy and habitual tonsillitis. Volcano plots showing differential gene expression in non-GC regions. CCND3 and HLA-C were upregulated in IgA nephropathy, while 16 genes, including TNFRSF17, were consistently downregulated (all adjusted p < 0.05). Few innate receptor genes (e.g., TLR4/5/7/9) showed notable differences, highlighting region-specific immune alterations.

In laser-microdissected GC plus mantle zones, KLRG1 and ZBTB16 showed higher expression in IgAN than in habitual tonsillitis (fold change: 3.21, p < 0.001; and 4.74, p = 0.02, respectively). Conversely, CCL18, FADD, and TNFRSF17 showed lower expression (fold change: -1.73, p = 0.01; -1.65, p = 0.02; and -1.54, p = 0.02, respectively) (**Figure 4**). Whole tonsil analysis revealed upregulation of KLRG1, ZBTB16, FKBP5, IL7R, and CXCR4 in IgAN (all adjusted p ≤ 0.02), with concomitant downregulation of HLA-DQB1, HLA-DQA1, HLA-DRB1, TNFRSF17, and CCL18 (adjusted p ≤ 0.01). In non-GC regions, CCND3 and HLA-C were upregulated in IgAN (fold change: 1.90, p < 0.001; and 1.55, p = 0.01), whereas 16 genes, including TNFRSF17, were consistently downregulated (all adjusted p < 0.05) (**Figure 5**). No notable differences were observed in TLR4, TLR5, TLR7, or TLR9 expression.

### Spatial distribution of KLRG1 expression

**Figure 6:**
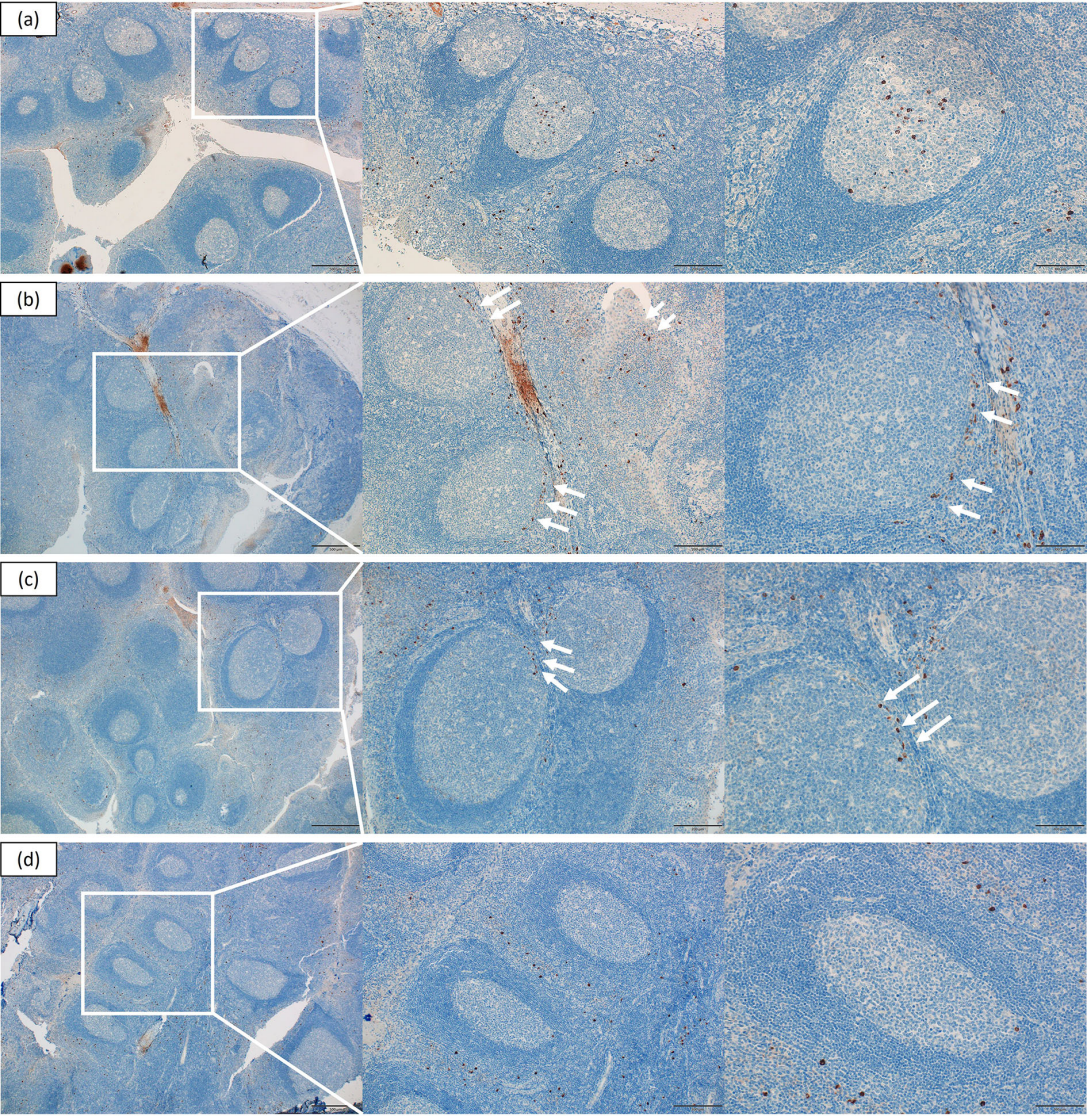
Spatial distribution of KLRG1 expression in tonsils from IgA nephropathy and habitual tonsillitis. Representative immunohistochemical staining of KLRG1 in IgA nephropathy **(a–c)** and habitual tonsillitis **(d)** (magnification ×40, ×100, ×200). In IgA nephropathy, KLRG1 was strongly localized within the GC plus mantle zone, with predominant signals in germinal centers, and with the most intense staining observed in patients with crescent formation **(a, b)**. In non-crescentic IgAN, KLRG1 was mainly restricted to marginal GC areas **(c)**. In habitual tonsillitis, KLRG1 staining was largely confined to non-GC regions **(d)**. Oxford classification for panels **(a–c)** was M0E0S0T0C1 **(a)**, M0E0S1T0C1 **(b)**, and M0E1S1T0C0 **(c)**. Arrows indicate KLRG1-positive staining.

Immunohistochemistry demonstrated distinct KLRG1 localization patterns between groups (**Figure 6**). In IgAN tonsils, KLRG1 staining was observed within the GC plus mantle zone and appeared more evident in germinal centers of patients with crescent formation, GC KLRG1 staining tended to be stronger in patients with crescent formation (3/3) than in those without (0/2), although the sample size limits definitive conclusions. In cases without crescents, KLRG1 was restricted to the marginal area of the germinal center. In contrast, in habitual tonsillitis, KLRG1 staining was largely confined to non-GC regions, with minimal germinal center involvement. Thus, GC-localized KLRG1 intensity appeared more evident in cases with crescent formation, suggesting a possible association between GC-localized KLRG1 expression and histological injury severity. However, given the small sample size, this observation should be interpreted with caution. These spatial differences suggested a possible region-specific pattern of KLRG1 expression in IgAN, although definitive conclusions regarding its relationship with glomerular injury severity cannot be drawn from this small cohort. These qualitative observations were supported by the quantitative analysis described below.

### Quantitative analysis of KLRG1 expression

**Figure 7:**
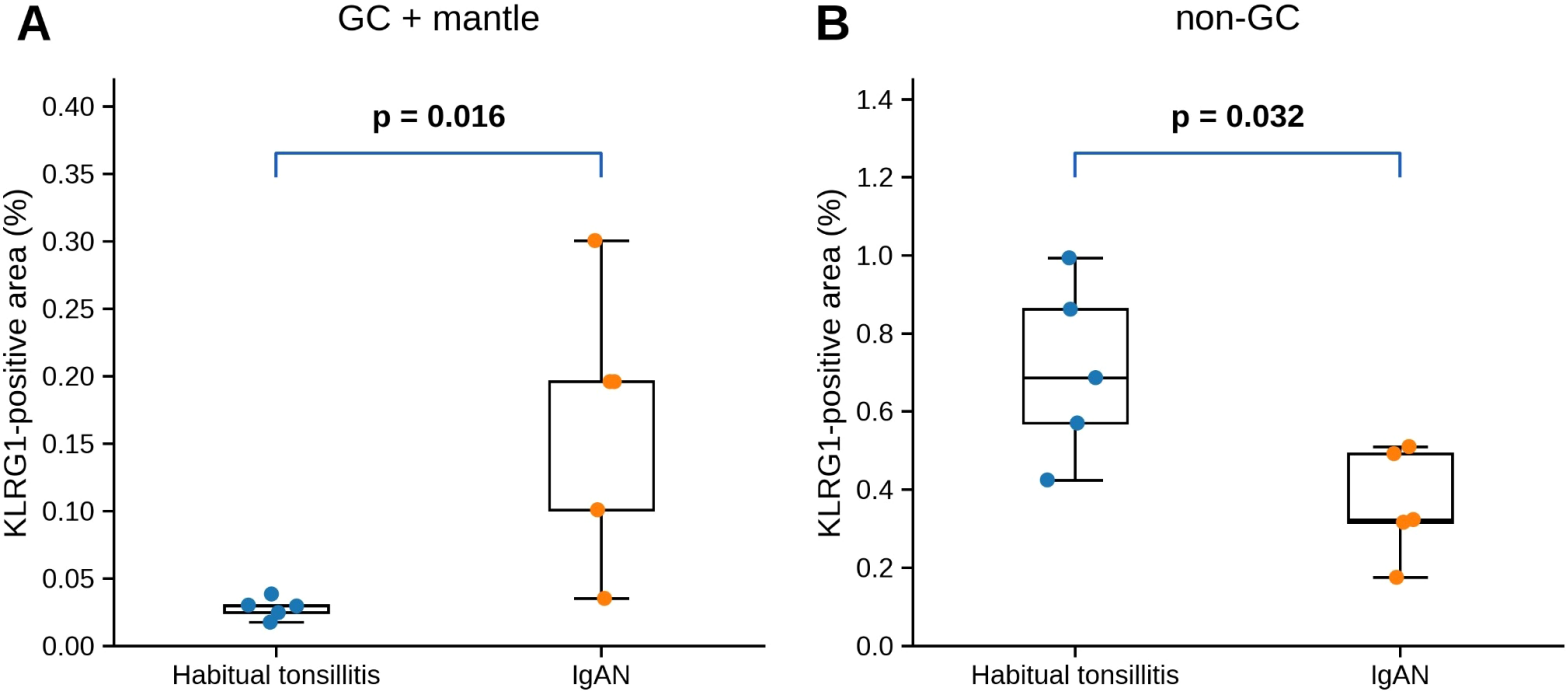
Quantitative analysis of KLRG1 immunohistochemistry. HT, habitual tonsillitis; IgAN, IgA nephropathy. **(A)** KLRG1-positive area (%) in the germinal center (GC) plus mantle zone. **(B)** KLRG1-positive area (%) in non-GC regions. Each dot represents an individual case (mean of multiple ROIs per case). Box-and-whisker plots show the median and interquartile range. KLRG1-positive area was quantified using ImageJ following color deconvolution (H DAB) and threshold-based segmentation, with a fixed threshold applied uniformly across all samples. ROIs with technical artifacts were excluded prior to averaging. Statistical comparisons were performed using the Mann–Whitney U test. P-values are indicated in the figure.

Quantitative image analysis demonstrated that KLRG1-positive area was significantly increased in the GC plus mantle zone in IgAN compared with habitual tonsillitis (p = 0.016) (**Figure 7A**). In contrast, in non-GC regions, KLRG1-positive area was significantly lower in IgAN compared with habitual tonsillitis (p = 0.032) (**Figure 7B**). Per-case analysis revealed consistent differences between groups, despite some intra-case variability. These findings suggest a region-specific pattern of KLRG1 distribution in the GC plus mantle zone in IgAN. Overall, digital pathology quantification showed a pattern consistent with the spatial transcriptomic findings in the GC plus mantle zone, while a different pattern was observed in non-GC regions.

### Sample-level NanoString expression analysis

**Figure 8:**
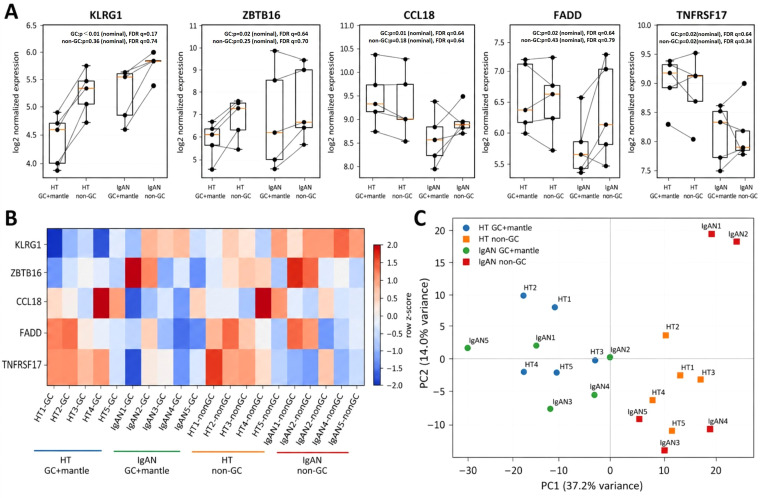
Sample-level NanoString expression analysis. HT, habitual tonsillitis; IgAN, IgA nephropathy. **(A)** Sample-level expression of key genes (KLRG1, ZBTB16, CCL18, FADD, and TNFRSF17) in GC plus mantle zone and non-GC regions. Each point represents an individual sample, and paired samples are connected by lines. Expression values are shown as log2-transformed normalized expression. Nominal p values and FDR-adjusted q values are shown for each gene. Although KLRG1 showed a nominally significant regional difference, it did not remain statistically significant after FDR correction (q = 0.17). **(B)** Heatmap of key genes showing row-wise z-score normalized expression (per gene) across samples. **(C)** Principal component analysis (PCA) of all genes based on log2-transformed normalized expression values. Samples are colored by disease group (HT vs IgAN) and shaped by tissue region (GC plus mantle zone vs non-GC).

Sample-level analysis of key genes demonstrated consistent expression patterns across individual cases. In the GC plus mantle zone, KLRG1 expression was consistently higher in IgAN compared with habitual tonsillitis across most samples, whereas differences in non-GC regions were less pronounced (**Figure 8A**). Although KLRG1 expression showed a nominally significant regional difference, this association did not remain statistically significant after FDR correction (q = 0.17). PCA revealed that samples were primarily separated according to tissue compartment (GC plus mantle zone vs non-GC regions), with additional separation by disease status (HT vs IgAN) (**Figure 8C**). Importantly, visual inspection did not suggest that the observed pattern was driven by a single obvious outlier. Heatmap analysis further demonstrated coherent and reproducible expression patterns within each group, supporting the robustness of the observed trends across samples (**Figure 8B**). Although KLRG1 expression in the GC plus mantle zone showed a marked increase with nominal statistical significance, this difference did not remain significant after multiple testing correction (FDR), indicating that the finding should be interpreted with caution.

## Discussion

In this study, we identified a germinal center (GC)–enriched immune signature in the tonsils of patients with immunoglobulin A nephropathy (IgAN), characterized by notable upregulation of KLRG1 and ZBTB16 and downregulation of CCL18, FADD, and TNFRSF17. These spatially resolved findings add to current understanding of IgAN pathogenesis, in which mucosal immune dysregulation drives nephritogenic IgA1 production (2–4), and provide new insights into the tonsillar immune microenvironment—a major mucosal site implicated in IgAN biology (5–8). Importantly, GC-localized KLRG1 expression appeared most prominent in patients with crescentic lesions, suggesting a possible association between this spatial immune pattern and histological injury severity; however, given the small sample size, this observation should be interpreted with caution and considered hypothesis-generating. Geographic variability in tonsillectomy efficacy has prompted long-standing debate regarding the role of tonsillar immunity in IgAN progression (9–16). Our demonstration that KLRG1 is enriched within GCs suggests that a GC environment populated by terminally differentiated or innate-like effector lymphocytes may reflect altered immune activity within the germinal center (2–4, 17–21). These findings raise the possibility that KLRG1 may serve as a candidate biomarker; however, further validation in larger cohorts is required, and the clinical implications remain to be determined. In this context, the observed KLRG1 expression pattern may reflect underlying differences in mucosal immune activation, although this interpretation remains speculative. Together, these findings are compatible with the concept of a potential KLRG1-enriched mucosal activation pattern in IgAN, characterized by the accumulation of terminally differentiated lymphocytes within germinal centers. Such GC-specific immune remodeling may reflect a mucosal immune pattern associated with IgAN, although its relationship with histological injury severity requires further validation, although this requires further validation.

KLRG1 is an inhibitory receptor expressed on terminally differentiated CD8^+^ T cells, NK cells, and tissue-resident effector populations (24–28). Its marked GC localization in IgAN is consistent with a model in which chronic or repetitive mucosal antigen exposure may expand differentiated lymphocyte subsets capable of modulating GC activity. Concurrent upregulation of ZBTB16, a key transcription factor for innate-like lymphocytes including NKT and MAIT cells (29), is broadly consistent with this interpretation. Reduced expression of TNFRSF17 (BCMA) (30) and CCL18 (31) further suggests attenuation of regulatory or plasma cell–supportive pathways, potentially shifting the GC milieu toward inflammatory or dysregulated selection pressures. Together, these molecular features indicate that IgAN GCs harbor an altered immune architecture that may be associated with altered immune processes within the germinal center (2–4, 18–21).

The tonsillar contribution to IgAN remains controversial, particularly in Western countries where tonsillectomy is not routinely practiced (15, 16). Recent morphometric studies demonstrated that IgAN tonsils exhibit significantly smaller lymphoid follicles and germinal centers compared with habitual tonsillitis controls, and that reduced follicular area was associated with heavier proteinuria and more advanced chronic histopathological changes in the kidney (32, 33). Our findings raise the possibility that GC KLRG1 enrichment may identify a subset of patients with a mucosal activation–associated pattern, which could help explain heterogeneous tonsillectomy responses observed across regions (15, 16). Pending confirmation in larger cohorts, KLRG1 and related GC signatures could help stratify patients by mucosal immunophenotype, thereby informing precision medicine strategies in IgAN. Although the cohort size in this study is modest due to the rarity of surgically obtained IgAN tonsils, the combination of laser microdissection and targeted Nanostring profiling enabled a level of spatial resolution that cannot be achieved by bulk tonsillar analysis. This approach captures niche-specific immune signatures with high analytical robustness, partly offsetting the limitations typically associated with small exploratory cohorts.

This study has several limitations. First, the sample size was small (5 vs 5), and the cross-sectional, single-center design limits generalizability. In particular, the small sample size limits the ability to draw definitive conclusions regarding the relationship between GC-localized KLRG1 expression and crescentic lesions. In addition, some observed associations, including KLRG1 expression differences, did not remain significant after multiple-testing correction and should therefore be interpreted cautiously. Second, all participants were Japanese, and ethnic differences in mucosal immune architecture cannot be excluded. Third, APRIL and BAFF, which are important mediators of mucosal IgA class switching, could not be evaluated in this study because these genes are not included in the Nanostring Human Immunology V2 Panel used for transcriptomic profiling. Therefore, mechanistic interpretations related to BAFF/APRIL-mediated B-cell selection should be interpreted with caution, and the involvement of this pathway could not be directly assessed in this study. Fourth, laser microdissection provides spatial resolution but does not define functional phenotypes of KLRG1^+^ cells. The cellular identity of KLRG1-positive cells could not be determined in this study. KLRG1 is not lineage-specific and may be expressed by multiple immune cell subsets, including CD8^+^ T cells, NK/NKT-like cells, and γδ T cells. Further studies using multiplex immunofluorescence or combined marker analyses are required to define the cellular composition of KLRG1-positive populations within the germinal center. Finally, FFPE tissue imposes inherent RNA degradation, although Nanostring technology mitigates this issue. These factors underscore that the findings should be interpreted as hypothesis-generating. Accordingly, validation in larger, independent cohorts is required. Despite the modest sample size, the use of laser microdissection coupled with Nanostring profiling provides high spatial resolution and analytical robustness, partly offsetting the limitations typically associated with small exploratory cohorts. Furthermore, functional validation of KLRG1^+^ lymphocyte subsets was beyond the scope of this study and remains an important direction for future work. Future studies incorporating functional characterization of KLRG1^+^ subsets and multi-omic spatial profiling will be essential to validate the biological relevance of this signature.

## Conclusion

We identified a GC-enriched KLRG1 expression pattern in IgAN tonsils. This spatial immune phenotype may reflect a mucosal activation–associated pattern potentially related to glomerular injury severity. However, because this was an exploratory, single-center study with a small sample size, these findings should be considered hypothesis-generating and require validation in larger, independent cohorts. This spatially resolved approach provides a framework for future studies investigating mucosal immune heterogeneity in IgAN.

## Data Availability

The datasets presented in this study can be found in online repositories. The names of the repository/repositories and accession number(s) can be found below: https://zenodo.org/records/17111571, ZENODO.
